# Prevalence of *Klebsiella pneumoniae* carbapenemase - and New Delhi metallo-beta-lactamase-positive *K. pneumoniae* in Sergipe, Brazil, and combination therapy as a potential treatment option

**DOI:** 10.1590/0037-8682-0064-2020

**Published:** 2020-05-11

**Authors:** Roberto Vivas, Silvio Santana Dolabella, Ana Andréa Teixeira Barbosa, Sona Jain

**Affiliations:** 1 Universidade Federal de Sergipe, Programa de Pós-Graduação em Biologia Parasitária, São Cristóvão, SE, Brasil.; 2 Universidade Tiradentes, Programa de Pós-Graduação em Biotecnologia Industrial, Aracaju, SE, Brasil.

**Keywords:** Carbapenemases, Polymyxin B, Amikacin, Tigecycline, Meropenem, Synergism

## Abstract

**INTRODUCTION:**

Carbapenem-resistant *Klebsiella pneumoniae* infection lacks treatment options and is associated with prolonged hospital stays and high mortality rates. The production of carbapenemases is one of the most important factors responsible for this multi-resistance phenomenon.

**METHODS:**

In the present study, we analyzed the presence of genes encoding carbapenemases in *K. pneumoniae* isolates circulating in one of the public hospitals in the city of Aracaju, Sergipe, Brazil. We also determined the best combination of drugs that display *in vitro* antimicrobial synergy. First, 147 carbapenem-resistant *K. pneumoniae* isolates were validated for the presence of blaKPC, *bla* GES, *bla* NDM, *bla* SPM, *bla* IMP, *bla* VIM, and *bla* OXA-48 genes using multiplex polymerase chain reaction. Thereafter, using two isolates (97 and 102), the role of double and triple combinational drug therapy as a treatment option was analyzed.

**RESULTS:**

Seventy-four (50.3%) isolates were positive for *bla* NDM, eight (5.4%) for *bla* KPC, and one (1.2%) for both *bla* NDM and *bla* KPC. In the synergy tests, double combinations were better than triple combinations. Polymyxin B and amikacin for isolate 97 and polymyxin B coupled with meropenem for isolate 102 showed the best response.

**CONCLUSIONS:**

Clinicians in normal practice use multiple drugs to treat infections caused by multi-resistant microorganism; however, in most cases, the benefit of the combinations is unknown. *In vitro* synergistic tests, such as those described herein, are important as they might help select an appropriate multi-drug antibiotic therapy and a correct dosage, ultimately reducing toxicities and the development of antibiotic resistance.

## INTRODUCTION

Antimicrobial resistance is a growing health problem and a serious threat to human health. It is estimated that by 2050, the infections caused by resistant microorganisms could be responsible for up to 10 million deaths annually worldwide ^1^ . According to a study conducted by the Centers for Disease Control and Prevention (CDC, USA), the hospitalization costs that are directly associated with multi-resistant microorganisms could reach $20 billion annually, besides the 35 billion dollars in additional indirect costs ^2^ .

*K. pneumoniae,* present as a common gut flora, can act as opportunistic pathogens, causing serious nosocomial infections in hospitalized and immunocompromised patients ^3^ . The emergence of antimicrobial resistance has complicated the management of infections caused by *K. pneumoniae* . A current major threat is the growing resistance to carbapenems as they are the last effective options available for antibiotic therapy against multi-resistant strains ^4^ *.* The rate of mortality associated with infections caused by Carbapenem-resistant *Klebsiella pneumoniae* may reach up to 75%, depending on the age and disease profile of the population analyzed ^5^ . Among the different factors associated with resistance to carbapenems, the production of enzymes that can degrade carbapenems are most prominent. Several types of carbapenemases have been described; however, from an epidemiological viewpoint, class A carbapenemases of the type *Klebsiella pneumoniae* carbapenemases (KPC) and class B carbapenemases of the type New Delhi metallo-beta-lactamases (NDM) are extremely important ^6^ ^,^ ^7^ . Both KPC and NDM have shown a rapid and widespread dissemination and their presence is often associated with multi-drug resistance ^8^ . Carbapenemases are frequently encoded on mobile genetic elements such as plasmids or transposons, which are responsible for their rapid transmission via horizontal gene transfer.

The shortage of treatment options for carbapenem-resistant *K. pneumoniae* infection often results in the use of combined therapies, which aim to achieve synergism via the combination of two or more drugs. Combination therapies can be a good alternative to monotherapy, which is associated with high mortality rates ^9^ . Many studies have reported a good response with combinatorial therapy; however, this is mainly achieved in an empiric manner without any pre-clinical evidence or *in vitro* analysis ^9^ ^-^ ^12^ .

Although most carbapenem-resistant *Enterobacteriaceae* demonstrate resistance to almost all antimicrobials, they have been found to have varying sensitivity to polymyxin (B or E), aminoglycosides, and tigecycline. Many studies have demonstrated the benefit of combining polymyxin with other antimicrobial agents, with polymyxin considered to be essential for the treatment of multi-resistant bacteria ^9^ ^,^ ^12^ ^,^ ^13^ . Although polymyxin was introduced more than 50 years ago, it remains one of the most important drug for the treatment of multi-resistant microorganism, despite being less efficacious and presenting more adverse effects compared with aminoglycosides and beta-lactams ^14^ . Polymyxin has been proposed to destabilize the bacterial cytoplasmic membrane, facilitating the action of other antimicrobials, such as meropenem and tigecycline ^15^ .

Owing to the reality and difficulty of treating *K. pneumoniae* producing carbapenemases, knowledge of genes coding for these carbapenemases in the circulating strains is important for epidemiological investigation, which is crucial for planning strategies to reduce the outbreak of infection and developing innovative therapeutic approaches. Nonetheless, a combination therapy with synergistic action can more adequately guide the therapeutic management of hospitalized patients. Data on the prevalence of carbapenem positive *K. pneumoniae* from the state of Sergipe are limited. The main aims of this study were to identify the genes coding for carbapenemases in multi-resistant *K. pneumoniae* isolates circulating in one of the public hospitals in the city of Aracaju, Sergipe, Brazil, and elucidate the best combination of drugs that display *in vitro* antimicrobial synergy.

## METHODS

### Isolates

One hundred and forty-seven (147) *K. pneumoniae* isolates belonging to the laboratory of microbiology of a public hospital in Northeast Brazil (Aracaju, Sergipe) were analyzed in this study. Identification and antimicrobial sensitivity tests were performed with an automated identification and susceptibility testing system (BD Phoenix, New Jersey, USA) using the NMIC-94 panel. The isolates were stored in Brain Heart Infusion broth with glycerol at -20 °C.

### Extraction of DNA and polymerase chain reaction (PCR)

Total DNA was isolated using the Wizard Genomic DNA Purification Kit (Promega, Brazil). The extracted DNA was quantified and stored at -20 °C. To detect the genes coding carbapenemases, multiplex PCR was conducted to target *bla* KPC *, bla* GES *, bla* NDM *, bla* SPM *, bla* IMP *, bla* VIM, and *bla* OXA-48 genes, which are usually reported to be present in *K. pneumoniae* isolates from Brazil ( Table 1 ) ^7^ ^,^ ^16^ . PCR was conducted in two different groups, containing 10 ng of total DNA, 12.5 µL of PCR mix (Taq DNA Polymerase Master Mix Red, Ampliqon, Denmark), and 0.1 µM of each primer (except *bla* IMP, where 0.2 µM was used) in a final volume of 25 µL. The first group contained primers for *bla* KPC *, bla* GES *, bla* NDM, and *bla* SPM, whereas the second group contained primers for *bla* IMP *, bla* VIM, and *bla* OXA-48. PCR was performed in 35 cycles at 95 ^o^ C for 50 s, 56 ^o^ C for 40 s, and 72 ^o^ C for 60 s. Amplified products were separated using 2% agarose and observed under ultraviolet light after staining with ethidium bromide.

**TABLE 1:** The primers used for multiplex polymerase chain reaction

PRIMER	SEQUENCE (5´ʹ → 3´ʹ)	SIZE (bp)	REFERENCE
*bla*_KPC_	**KPC F** / TGT CAC TGT ATC GCC GTC TAG	880	(16)
	**KPC R** / TTA CTG CCC GTT GAC GCC CAA TCC		
			
*bla*_GES_	**GES F** / ATG CGC TTC ATT CAC GCA C	591	(16)
	**GES R** / CTA TTT GTC CGT GCT CAG G		
			
*bla*_IMP_	**IMP F** / GAG TGG CTT AAT TCT CRA TC	120	(7)
	**IMP R** / AAC TAY CCA ATA YRT AAC		
			
*bla*_VIM_	**VIM F** / GAT GGT GTT TGG TCG CAT A	390	(16)
	**VIM R** / CGA ATG CGC AGC ACC AG		
			
*bla*_SPM_	**SPM F** / AAA TCT GGG TAC GCA AAC G	270	(16)
	**SPM R** / AGA TTA TCG GCT GGA ACA GG		
			
*bla*_NDM_	**NDM F** / TTG GCC TTG CTG TCC TTG	82	(7)
	**NDM R** / ACA CCA GTG ACA ATA TCA CCG		
			
*bla*_OXA-48_	**OXA48 F** / TTG GTG GCA TCG ATT ATC GG	743	(16)
	**OXA48 R** / GAG CAC TTC TTT TGT GAT GGC		

### Checkerboard

Checkerboard ^17^ was carried out using isolates 97 and 102 with double or triple combinations of polymyxin B, amikacin, tigecycline and meropenem, which are generally used in clinical practice to treat infections caused by multi-resistant *K. pneumoniae* . Briefly, the minimum inhibitory concentration (MIC) value of the four antibiotics against each isolate was measured using broth microdilution (BMD) ^18^ . Thereafter, the synergism between different combinations was evaluated according to the fractional inhibitory concentration index (FICI), which was calculated as the sum of the FICs of individual drugs. The FICs of individual drugs were calculated as follows: FIC of a drug = MIC of the drug in combination/MIC of drug alone. The FICIs were interpreted as follows: synergy, FICI ≤0.5; additivity, FICI >0.5 to ≤1; indifference, FICI >1 to ≤4; antagonism, FICI >4.

The isolates were seeded in solid media (blood agar) and incubated overnight at 36 °C for 20 h under aerobic conditions. Microbial suspensions were prepared in sterile saline with an optical density of McFarland standard scale 0.5. A total of 50 μL of seven different dilutions of meropenem, polymyxin B, tigecycline, and amikacin (in double combinations) were placed in each well of a 96-well microplate, with 90 μL of Muller-Hinton broth and 10 μL of the isolate. The double combinations tested were polymyxin B + meropenem, polymyxin B + tigecycline, and polymyxin B + amikacin. For the triple combinations, 50 μL of 11 different dilutions of meropenem, and seven different dilutions of tigecycline and amikacin were used. Polymyxin B was added as a fixed concentration depending on its MIC against the isolate analyzed. A total of 40 μL of Muller-Hinton broth and 10 μL of the isolate suspension was later added. The triple combinations tested were polymyxin B + meropenem + tigecycline and polymyxin B + meropenem + amikacin. The final volume was maintained at 200 μL for the double and triple combinations. The 96-well microplates were incubated at 36 °C for 20 h under aerobic conditions and were read using an EPOCH equipment (Biotek, Winooski, Vermont, USA). The experiments were conducted in triplicate via three independent experiments. The results are expressed as means plus standard deviations.

## RESULTS

One hundred and forty-seven isolates identified as *K. pneumoniae* and presenting varied resistance to carbapenems (ertapenem, imipenem, and meropenem; Supplementary Information) were tested for the presence of genes coding carbapenemases ( *bla* KPC *, bla* GES *, bla* NDM *, bla* SPM *,bla* IMP *, bla* VIM, and *bla* OXA-48). Eighty-three (56.5%) of these isolates were positive for the presence of one or more of the genes analyzed: 74 (50.3%) were positive for *bla* NDM, 8 (5.4%) were positive for *bla* KPC, and 1 isolate (1.2%) was positive for both *bla* NDM and *bla* KPC.

The MIC of ertapenem was >0.5 μg/mL against all isolates, unlike that of imipenem and meropenem, which had different sensitivities (Supplementary Information). The isolates positive for *bla* NDM or *bla* KPC showed high resistance to the carbapenems tested (Supplementary Information). Only 1.3% of the isolates that were positive for *bla* NDM showed sensitivity to imipenem and meropenem. Conversely, 14.3% of the *bla* KPC-positive isolates showed sensitivity to imipenem and meropenem. Compared to other classes of antimicrobials, polymyxin presented an excellent activity against the isolates encoding *bla* KPC with 100% sensitivity. Nonetheless, 97.4% of the *bla* NDM positive isolates were found to be sensitive to polymyxin ( Table 2 ).

**TABLE 2:** Sensitivity profile for the NDM- ( *n=* 74) and KPC ( *n=* 8)-positive isolates.

	***bla* NDM**	***bla* KPC**
Antibiotics	(%)	(%)
Amikacin	73.7	71.4
Tigecycline	46.1	42.9
Polymyxin B	97.4	100
Meropenem	1.3	14.3
Imipenem	1.3	14.3
Ertapenem	0	0

**blaNDM:** gene encoding New Delhi metallo-beta-lactamase enzyme; **blaKPC:** gene encoding Klebsiella pneumoniae carbapenemase enzyme.

Isolates 97 and 102, which displayed different sensitivity profiles (Supplementary Information), were used for synergistic tests. The MICs of drugs against these isolates were measured by BMD as well as the automated susceptibility testing system (BD Phoenix), as shown in Table 3 . The results for these isolates using double or triple combinations of polymyxin B, amikacin, tigecycline, and meropenem are shown in Table 4 . For isolate 97, the FICI values showed synergism for the double combinations of polymyxin and amikacin and for the triple combination of amikacin, meropenem, and polymyxin. For the other combinations tested, the effect was additive. For isolate 102, the double and triple combinations tested showed an additive effect.

**TABLE 3:** Minimum inhibitory concentration values (μg/mL) of drugs against isolates 97 and 102 determined using broth microdilution (BMD) and the automated system.

	Isolate 97		Isolate 102	
	BMD	Automated system	BMD	Automated system
Polymyxin B	1 (S)	≤ 1 (S)	0.25 (S)	≤ 1 (S)
Tigecycline	0.5 (S)	≤ 1 (S)	2 (I)	4 (R)
Amikacin	8 (S)	≤ 8 (S)	32 (I)	32 (I)
Meropenem	8 (R)	> 8 (R)	64 (R)	> 8 (R)

**S:** sensitive; **I:** intermediate; **R:** resistant.

**TABLE 4:** Checkerboard results presented as fractional inhibitory concentration index.

	Antimicrobial combinations				
	POL+AMI	POL+MER	POL+TGC	AMI+MER+POL	TGC+MER+POL
Isolate 97	**0.34 (0.05)**	0.63 (0.18)	**0.75 (0.00)**	**0.33 ( 0.02)**	**0.66 (0.03)**
Isolate 102	**0.62 (0.00)**	**0.53 (0.05)**	**0.90 (0.23)**	**0.83 (0.13)**	**0.87 (0.17)**

**POL:** polymyxin B; **AMI:** amikacin; **TGC:** tigecycline; **MER:** meropenem, values in parentheses represent standard deviations.

## DISCUSSION

**FIGURE 1:**
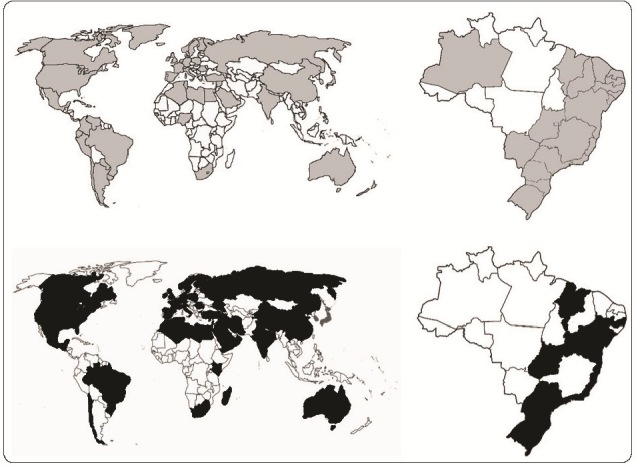
Distribution of *bla* KPC (gray) and *bla* NDM (black) in *K. pneumoniae* across the world (left) and in Brazil (right). The references used to generate this figure have been listed in the Supplementary Information (SI 2) .

Carbapenems display broad-spectrum antibacterial activity. In addition, they have a unique structure defined by a carbapenem coupled to a β-lactam ring, which confers protection against most β lactamases. Carbapenems are thus one of the most reliable drugs for treating bacterial infections. The first reports of carbapenemases occurred in the 1980s and their rapid spread worldwide constitutes a major public health concern globally ^19^ . *K. pneumoniae* encoding KPC was first reported in North Carolina, USA in 2001. Since 2001, approximately 20 KPC subtypes have been reported among different gram-negative organisms from different regions of the world. KPC is considered endemic in the US, China, Italy, Poland, Greece, Israel, Brazil, Argentina, and Colombia ^20^ ^-^ ^22^ . In Brazil, it is the most common carbapenem described in *Enterobacteriaceae* . The first report of KPC in Brazil was from the city of Recife, Pernambuco in 2006. However, today, KPC positive isolates ( Figure 1 ) have been reported in almost all parts of Brazil ^23^ ^-^ ^25^ .

NDM-1 was first described in *K. pneumoniae* isolated from an Indian patient in Sweden in 2008 ^26^ . Today, at least eight NDM variants are known and reported from all continents worldwide ^22^ . Five years following its discovery in 2008, NDM was identified in Brazil ^27^ ; this incidence differed from that of KPC, which was reported in 1996 in the USA and then approximately 10 years later in Brazil in 2006 ^23^ ^,^ ^28^ ^,^ ^29^ .

Similar to KPC, NDM has been associated with multi-resistance and has been reported from various Brazilian states and in different gram-negative species ^30^ . Approximately 50% of the isolates analyzed in this study were positive for NDM, followed by KPC (8%). These results demonstrate the rapid dissemination capacity of the NDM-positive isolates and indicate an urgent need for alternative therapy for infections caused by these multi-resistant isolates.

In the state of Sergipe, where this study was conducted, studies on multi-resistant carbapenemases-producing *K. pneumoniae* are limited. To our knowledge, this study is one of the first scientific reports describing *K. pneumoniae* isolates with *bla* KPC from this state. The first case of an *Enterobacteriaceae* ( *K. pneumoniae and Citrobacter freundii* ) with *bla* NDM in this region was reported in 2015 in the neighboring state of Bahia ^27^ ^,^ ^31^ . Additionally, a recently published article (in 2019) reported the first case of NDM in the state of Sergipe from *K. pneumoniae* samples collected between 2012 and 2015 ^30^ . In our study, one of the isolates (isolate 98) showed the presence of both *bla* NDM and *bla* KPC. KPC and NDM enzymes are rarely reported in a single strain, and the co-production of these carbapenemases in a single strain could confer high resistance to carbapenems ^32^ ^,^ ^33^ .

Of the 147 carbapenem-resistant isolates analyzed by this study, 64 (43.5%) were negative for all the genes analyzed. This can be justified as these strains act via other mechanisms of resistance, such as mechanisms involving the presence or absence of porins/efflux pumps or a gene that has not been investigated in this study. A major challenge in the control of multi-resistant microorganisms (apart from the lack of treatment options) is the identification and emergence of new resistance mechanism. One such example is the recent identification of a new carbapenem Brazilian *Klebsiella* carbapenem in the city of São Paulo, Brazil ^34^ . Another challenge is associated with the emergence of microorganisms with atypical sensitivity profiles. For example, the identification of *K. oxytoca* isolates sensitive to ceftriaxone and cefepime (3rd and 4th generation cephalosporins, respectively) but resistant to carbapenems (without any genes identified for carbapenemases and ESBL) described by a group of researchers in the United States ^35^ .

In our study, ertapenem proved to be a good marker for the suspected production of carbapenemases as ertapenem had an MIC of >0.5 μg/mL for the isolates analyzed herein. Prior studies have demonstrated the importance of determining the sensitivity to ertapenem as it is the most sensitive indicator for detecting carbapenemases. However, resistance to this carbapenem is not a direct indicator for the production of carbapenemases ^36^ . Other impermeability mechanisms such as the loss of porins, efflux pumps, and an association with enzymes, such as ESBL and AmpC, may decrease the activity of ertapenem ^37^ .

Polymyxins (B and E) are widely used as one of the last therapeutic resorts to treat bacterial resistant to carbapenems. Indeed, most of the isolates analyzed in this study displayed sensitivity to polymyxin. However, resistance to polymyxin has been observed only recently and has already been identified in several carbapenem-resistant *K. pneumoniae* . Many mechanisms associated with chromosomal genes have been described ^14^ . In 2016, a plasmid mediated MCR-1 gene could confer resistance to polymyxins and transfer to other gram-negative species was reported ^38^ . The global distribution of easily transferable MCR-1 gene in bacterial strains poses a substantial health concern ^39^ .

In clinical practice, empirical antibiotic therapy is extremely important as the delay in appropriate antimicrobial therapy might result in unfavorable clinical evolution, especially in patients with severe infection. Several factors are involved in the selection of a good empirical antibiotic therapy and among these, knowledge of the most probable microorganism might be most important. Accordingly, the results of the antimicrobial susceptibility profile for the hospital microbiota can serve as an important guide ^13^ .

Among the drugs evaluated in this study, only polymyxin offered security as an empirical therapy. Further, as 70% of the isolates were sensitive to amikacin ( Supplementary Information ), it can also be employed as a therapeutic option, despite the ~30% resistance observed.

Carbapenem-resistant *K. pneumoniae* is usually resistant to most beta-lactams. As a result, the treatment options are limited to polymyxins, tigecycline, and aminoglycosides ^40^ . Because of the therapeutic failures associated with monotherapy, combination therapy is usually recommended for the treatment of serious infections caused by multi-resistant microorganisms. However, clinical evidence of this strategy is currently limited and randomized trials are needed to highlight more effective drug combinations ^22^ .

In the present study, checkerboard was used to validate the double and triple combinations of polymyxin B, amikacin, tigecycline, and meropenem with isolates 97 and 102. Polymyxin B, amikacin, tigecycline, and meropenem are generally used in clinical practice to treat infections caused by multi-resistant *K. pneumoniae* . Isolate 97 showed a synergistic effect when polymyxin B and amikacin were combined together (FICI of 0.34). Similarly, when used in the triple combination (polymyxin B + amikacin+ meropenem), synergism was observed, with an FICI of 0.33, thereby displaying a minimal difference between the double and triple combinations. As a result, the double combination was found to have a response effective as the triple combination. The use of meropenem as a third drug for antibiotic therapy should thus be re-assessed in the combination therapy. Additionally, these *in vitro* results require validation through further *in vivo* studies to better confirm the success of double-drug therapy relative to triple-combination therapy.

The other combinations analyzed for isolate 97 demonstrated an additive effect, with the polymyxin B and meropenem combination displaying a lower FICI of 0.63 than the polymyxin B and tigecycline combination (FICI of 0.75). Additionally, this double combination of polymyxin B + meropenem was found to have an FICI (0.63) lower than that of the triple combination of polymyxin B + meropenem + tigecycline with FICI of 0.66, thereby showing that the combination with tigecycline may be less active than the combinations without tigecycline.

No synergistic action was observed for isolate 102 in the combinations analyzed. The lowest FICI of 0.53 was obtained for the combination, polymyxin B + meropenem, followed by 0.62 for polymyxin B + amikacin and 0.90 for polymyxin B + tigecycline. In the triple combinations, the FICIs found were superior to the double combination of polymyxin B + meropenem. In a study conducted in Germany, a similar result was obtained when the triple and double combinations using meropenem, tigecycline, and colistin were analyzed. In fact, the FICI of some of the triple combinations was superior to that of the double combinations ^15^ . For isolate 102, double combinations had a better result than the triple combinations, at least *in vitro* . Thus, double combinations with polymyxin B and amikacin for isolate 97 and polymyxin B plus meropenem for isolate 102 had the lowest FICI.

As each country, region, or health institute has specific microbiota that are dependent on innumerous factors specific to the respective environment, the results of the synergistic tests are also specific to the isolate used in the analysis, which complicates the generalizability of such results. The differences between MICs and other associated mechanisms, such as the loss of porins, efflux pump, and the production of other beta-lactamases, such as ESBL and AmpC, may justify the different checkerboard results obtained between isolates 97 and 102.

As there have been no new developments in antibiotics for decades, it is estimated that by 2050, there will be no effective antibiotics available for the treatment of infectious diseases ^1^ . Owing to the alarming increase in multi-resistant bacteria and the lack of new drugs for their treatment, combination therapy is presently the only available option. In regular practice, clinicians are administering multiple drugs to treat these infections. However, in most cases, these combinations are administered without any knowledge of their benefit. *In vitro* synergistic tests, such as those presented herein, are very important as they may serve as a guide for the selection of appropriate antibiotic therapies and for the achievement of favorable clinical outcomes. Additionally, these tests may assist clinicians in the administration of lower and effective doses, thereby reducing the toxicities associated with the use of multiple drugs and slowing the development of antibiotic resistance.
