# Supplementary material for: Prevalence of Klebsiella pneumoniae carbapenemase - and New Delhi metallo-beta-lactamase-positive K. pneumoniae in Sergipe, Brazil, and combination therapy as a potential treatment option
Source: Rev Soc Bras Med Trop. 2020 May 11;53:e20200064. doi: 10.1590/0037-8682-0064-2020 (PMC7269519; doi:10.1590/0037-8682-0064-2020)
Supplement: Supplementary file 1 [file 1678-9849-rsbmt-53-e20200064-suppl1.pdf]

SI 2: Reference used to generate figure 1

1. Du J, Li P, Liu H, Lü D, Liang H, Dou Y. Phenotypic and molecular characterization of multidrug resistant klebsiella pneumoniae isolated from a university teaching hospital, China. PLoS One. 2014;9(4).
2. Monteiro J, Widen RH, Pignatari ACC, Kubasek C, Silbert S. Rapid detection of carbapenemase genes by multiplex real-time PCR. J Antimicrob Chemother. 2012;67(4):906–9.
3. Faria-Junior C. NDM-Producing Enterobacteriaceae Strains among Hospitals in Brasília, Brazil. J Microbiol Exp. 2016;3(2).
4. Ageevets VA, Partina I V, Lisitsyna ES, Ilina EN, Lobzin Y V, Shlyapnikov SA, et al. Emergence of carbapenemase-producing Gram-negative bacteria in Saint Petersburg, Russia. Int J Antimicrob Agents. 2014;44(2):152–5.
5. Horna G, Velasquez J, Fernández N, Tamariz J, Ruiz J. Characterisation of the first KPC-2-producing Klebsiella pneumoniae ST340 from Peru. J Glob Antimicrob Resist. 2017;9:36–40.
6. Rosabel Dienstmann ; Simone Ulrich Picoli ; Gabriela Meyer ; Tiago Schenkel ; Juçara. Avaliação fenotípica da enzima Klebsiella pneumoniae carbapenemase (KPC) em Enterobacteriaceae de ambiente hospitalar. J Bras Patol Med Lab. 2010;46(1):23–7.
7. Escandón-vargas K, Reyes S, Gutiérrez S, Virginia M, Escandón-vargas K. The epidemiology of carbapenemases in Latin America and the Caribbean. Expert Rev Anti Infect Ther. 2017;15(3):277–97.
8. Carrasco-anabalón S, Orlando C, Neto C, Paula A, Carvalho-assef DA, Lima CA, et al. International Journal of Infectious Diseases Introduction of NDM-1 and OXA-370 from Brazil into Chile in strains of Klebsiella pneumoniae isolated from a single patient. Int J Infect Dis. 2019;81:28–30.
9. Mohammed Y, Zailani SB, Onipede AO. Characterization of KPC, NDM and VIM

Type Carbapenem Resistance Enterobacteriaceae from North Eastern, Nigeria. J Biosci Med. 2015;03(11):100–7.

10. Fehlberg LCC, Carvalho AMC, Campana EH, Gontijo-Filho PP, Gales AC. Emergence of Klebsiella pneumoniae-producing KPC-2 carbapenemase in Paraíba, Northeastern Brazil. Brazilian J Infect Dis. 2012;16(6):577–80.
11. Chang MR, Biberg CA, Lopes FA, Tetila AF, Carlos A, Pignatari C. The first report of infection with Klebsiella pneumoniae carrying the bla kpc gene in State of Mato Grosso do Sul , Brazil. Rev Soc Bras Med Trop. 2013;46(1):114–5.
12. Vieira N, Filho HF, Alex D, Joaquim A, Calvalcante W, Garcia DDO, et al. First report of a NDM-producing Providencia rettgeri strain in the state of São Paulo. Brazilian J Infect Dis. 2013;19(6):675–6.
13. Scavuzzi AML, Firmo EF, Oliveira ÉM De, Lopes AC de S. Emergence of bla NDM-1 associated with the aac(6')-Ib-cr, acrB, cps, and mrkD genes in a clinical isolate of multi-drug resistant Klebsiella pneumoniae from Recife-PE, Brazil. Rev Soc Bras Med Trop. 2019;52(March):0–3.
14. Tuon FF, Rocha JL, Toledo P, Arend LN, Dias CH, Leite TM, et al. Risk factors for KPC-producing Klebsiella pneumoniae bacteremia. Brazilian J Infect Dis. 2012;16(5):416–9.
15. Marques AS, Moraes EP, Júnior MAA, Moura AD, Neto VFA, Neto RM, et al. Rapid discrimination of klebsiella pneumoniae carbapenemase 2 – producing and non-producing klebsiella pneumoniae strains using near-infrared spectroscopy (NIRS) and multivariate analysis. Talanta. 2015;134:126–31.
16. Pereira PS, de Araujo CFM, Seki LM, Zahner V, Carvalho-Assef APD, Asensi MD. Update of the molecular epidemiology of KPC-2-producing Klebsiella pneumoniae in Brazil: spread of clonal complex 11 (ST11, ST437 and ST340). J Antimicrob Chemother. 2013;68(2):312–6.
17. Ferreira AM, Mondelli AL, Campos e Silva JGS, Javaroni E, Gambarini PS, de Oliveira Garcia D, et al. First report of a clinical isolate of New Delhi metallo-β-lactamase-producing Klebsiella pneumoniae in Brazil. J Hosp Infect. 2016;94(1):73–4.

18. Aires CAM, Pereira PS, de Araujo CFM, Chagas TPG, Oliveira JCR, Buonora SN, et al. Multiclonal Expansion of Klebsiella pneumoniae Isolates Producing NDM-1 in Rio de Janeiro, Brazil. Antimicrob Agents Chemother. 2017;61(4):1–6.
19. Saito R, Takahashi R, Sawabe E, Koyano S, Takahashi Y, Shima M, et al. First Report of KPC-2 Carbapenemase-Producing Klebsiella pneumoniae in Japan. Antimicrob Agents Chemother. 2014;58(5):2961–3.
20. Silva IR da, Aires CAM, Conceição-Neto OC, de Oliveira Santos IC, Ferreira Pereira N, Moreno Senna JP, et al. Distribution of Clinical NDM-1-Producing Gram-Negative Bacteria in Brazil. Microb Drug Resist. 2019;25(3):394–9.
