# Supplementary material for: Prevalence of Klebsiella pneumoniae carbapenemase - and New Delhi metallo-beta-lactamase-positive K. pneumoniae in Sergipe, Brazil, and combination therapy as a potential treatment option
Source: Rev Soc Bras Med Trop. 2020 May 11;53:e20200064. doi: 10.1590/0037-8682-0064-2020 (PMC7269519; doi:10.1590/0037-8682-0064-2020)
Supplement: Supplementary file 2 [file 1678-9849-rsbmt-53-e20200064-suppl2.pdf]

SI 1: MIC values of drugs against 147 *K. pneumoniae* isolates determined using the automated system

SAMPLES	AMI	GEN	CFP	CFO	CIP	COL	ETP	IMI	MER	TGC
1	≤8	≤2	>16	>16	≤0.5	≤1	>4	2	8	≤1
2	≤8	≤2	≤1	>16	>2	≤1	>4	2	8	>4
3	16	>8	≤1	>16	>2	≤1	>4	2	8	2
4	≤8	>8	>16	>16	≤0.5	≤1	>4	>8	8	2
5	≤8	>8	>16	>16	>2	≤1	>4	>8	>8	2
6	≤8	>8	≤1	>16	>2	≤1	>4	2	4	4
7	32	>8	≤1	>16	>2	≤1	>4	>8	>8	4
8	32	>8	≤1	>16	>2	≤1	>4	2	4	4
9	32	>8	≤1	>16	>2	≤1	>4	>8	>8	4
10	16	>8	≤1	>16	>2	≤1	>4	>8	>8	4
11	16	>8	>16	>16	>2	≤1	>4	>8	>8	4
12	32	>8	>16	>16	>2	≤1	>4	>8	>8	4
14	16	>8	≤1	>16	>2	≤1	>4	4	8	2
15	32	4	>16	>16	>2	≤1	>4	2	8	≤1
16	32	>8	>16	>16	>2	≤1	>4	2	8	2
17	≤8	>8	>16	>16	>2	≤1	>4	4	8	4
18	32	>8	>16	>16	>2	≤1	>4	2	8	2
19	≤8	≤2	>16	>16	>2	≤1	>4	4	8	4
20	≤8	>8	>16	>16	>2	≤1	>4	>8	>8	≤1
22	>32	≤2	>16	>16	≤0.5	≤1	>4	>8	>8	≤1
23	≤8	>8	>16	>16	>2	>4	>4	>8	>8	2
24	32	>8	>16	>16	>2	≤1	>4	8	>8	2
25	≤8	>8	>16	>16	>2	>4	1	≤1	≤1	2
26	≤8	>8	>16	>16	>2	>4	>4	2	4	4
27	>32	>8	>16	>16	>2	≤1	>4	>8	>8	4
28	≤8	≤2	-	>16	>2	≤1	>4	≤1	2	>4
29	32	>8	>16	>16	>2	≤1	>4	>8	>8	4
30	≤8	>8	>16	>16	≤0.5	≤1	>4	2	2	2

31	≤8	>8	>16	8	>2	≤1	1	≤1	≤1	2
32	≤8	>8	>16	>16	>2	≤1	4	≤1	≤1	4
33	≤8	>8	≤1	>16	>2	>4	>4	2	4	2

SI 1 (continued)

SAMPLES	AMI	GEN	CFP	CFO	CIP	COL	ETP	IMI	MER	TGC
34	32	>8	≤1	>16	>2	≤1	>4	4	8	2
35	>32	>8	>16	>16	>2	≤1	>4	>8	>8	4
36	≤8	>8	>16	>16	≤0.5	≤1	>4	>8	>8	2
37	≤8	≤2	>16	>16	>2	≤1	>4	2	4	>4
38	32	>8	≤1	>16	>2	≤1	>4	>8	>8	4
39	≤8	>8	>16	>16	>2	>4	>4	4	>8	2
40	32	4	>16	16	≤0.5	≤1	>4	8	8	2
42	≤16	≤4	>8	>16	>2	≤2	>1	≤1	2	>2
43	≤16	>8	>8	>16	>2	4	>1	8	>8	≤1
44	≤16	≤4	>8	>16	≤1	≤2	>1	4	8	2
45	≤16	>8	≤1	>16	>2	≤2	>1	≤1	≤1	≤1
46	≤16	>8	>8	>16	>2	≤2	>1	≤1	≤1	≤1
47	≤16	>8	>8	>16	>2	≤2	>1	2	4	≤1
48	32	>8	>8	>16	>2	-	>1	≤1	≤1	2
49	≤16	>8	>8	>16	>2	4	>1	8	>8	≤1
50	≤8	≤2	>16	≤4	2	≤1	>4	≤1	≤1	≤1
51	>32	>8	≤1	>16	>2	≤1	>4	>8	>8	4
52	≤8	>8	>16	>16	>2	>4	>4	4	>8	≤1
53	≤8	>8	>16	>16	>2	>4	>4	4	>8	≤1
54	32	>8	≤1	>16	>2	≤1	>4	>8	>8	4
55	≤8	>8	≤1	>16	>2	≤1	>4	>8	>8	≤1
56	≤8	≤2	-	>16	>2	≤1	>4	≤1	≤1	>4
58	≤8	>8	>16	>16	>2	≤1	>4	≤1	2	4
60	≤8	>8	≤1	>16	>2	≤1	>4	2	2	2
61	≤8	≤2	-	>16	>2	≤1	1	≤1	≤1	2
62	≤8	>8	-	8	>2	≤1	1	≤1	≤1	4

64	≤8	>8	≤1	>16	>2	≤1	>4	2	8	≤1
65	32	>8	>16	>16	>2	≤1	>4	>8	>8	4
66	32	>8	≤1	>16	>2	≤1	>4	>8	>8	4
67	16	4	>16	16	1	≤1	>4	>8	8	≤1
68	≤8	≤2	≤1	>16	>2	≤1	>4	2	8	≤1

SI 1 (continued)

SAMPLES	AMI	GEN	CFP	CFO	CIP	COL	ETP	IMI	MER	TGC
69	32	>8	≤1	>16	>2	≤1	>4	>8	>8	4
70	≤8	>8	>16	>16	>2	>4	>4	4	8	2
71	≤8	>8	>16	>16	>2	≤1	>4	>8	>8	4
72	32	>8	≤1	>16	>2	≤1	>4	>8	>8	4
73	32	>8	16	16	>2	≤1	>4	2	4	2
74	≤8	>8	>16	>16	>2	≤1	>4	≤1	≤1	≤1
75	≤8	>8	>16	>16	>2	≤1	>4	2	8	2
76	≤8	>8	>16	>16	≤0.5	≤1	>4	>8	>8	≤1
77	≤8	>8	>16	>16	≤0.5	≤1	>4	>8	>8	≤1
78	≤8	>8	>16	>16	>2	≤1	>4	2	4	≤1
79	≤8	>8	>16	>16	>2	≤1	>4	>8	>8	2
80	≤8	>8	>16	>16	>2	≤1	>4	2	4	≤1
81	≤8	>8	-	>16	>2	≤1	>4	≤1	2	≤1
82	32	>8	-	>16	>2	≤1	>4	8	>8	≤1
83	≤8	>8	>16	>16	>2	≤1	>4	>8	8	≤1
84	≤8	>8	>16	>16	≤0.5	≤1	>4	>8	>8	≤1
87	≤8	≤2	>16	>16	≤0.5	≤1	>4	>8	>8	≤1
88	≤8	>8	>16	>16	>2	≤1	>4	>8	>8	≤1
89	≤8	>8	>16	>16	≤0.5	≤1	>4	>8	8	≤1
91	≤8	>8	>16	>16	>2	≤1	>4	8	>8	2
92	16	≤2	≤1	>16	>2	≤1	>4	4	8	≤1
93	16	≤2	>16	>16	≤0.5	≤1	>4	>8	>8	≤1

94	≤8	>8	>16	>16	>2	≤1	>4	>8	>8	2
95	≤8	>8	>16	>16	>2	≤1	>4	>8	>8	≤1
96	≤8	≤2	>16	>16	≤0.5	≤1	>4	>8	>8	≤1
97	≤8	≤2	>16	>16	≤0.5	≤1	>4	>8	>8	≤1
98	≤8	>8	>16	>16	≤0.5	≤1	>4	>8	>8	2
99	≤8	>8	>16	>16	≤0.5	≤1	>4	>8	>8	2
100	≤8	>8	>16	>16	2	≤1	>4	>8	>8	≤1
101	32	>8	≤1	>16	>2	≤1	>4	>8	>8	4
102	32	>8	≤1	>16	>2	≤1	>4	>8	>8	4

SI 1 (continued)

SAMPLES	AMI	GEN	CFP	CFO	CIP	COL	ETP	IMI	MER	TGC
104	32	>8	>16	>16	>2	≤1	>4	>8	>8	4
105	≤8	≤2	>16	>16	≤0.5	≤1	>4	>8	8	≤1
106	≤8	>8	>16	>16	>2	≤1	>4	>8	4	≤1
107	≤8	>8	>16	>16	>2	≤1	>4	8	4	≤1
108	<8	>8	>16	>16	≤0.5	≤1	>4	>8	8	4
109	≤8	>8	>16	>16	≤0.5	≤1	>4	>8	>8	≤1
110	≤8	>8	>16	>16	≤0.5	≤1	>4	>8	8	2
111	≤8	>8	>16	>16	>2	≤1	>4	8	8	2
112	≤8	>8	>16	>16	>2	≤1	>4	>8	8	2
113	32	>8	>16	>16	>2	≤1	>4	>8	>8	4
114	32	>8	≤1	>16	>2	≤1	>4	>8	>8	4
116	≤8	>8	>16	>16	>2	≤1	>4	>8	>8	≤1
118	≤8	>8	>16	>16	≤0.5	≤1	>4	8	8	≤1
119	16	>8	>16	>16	>2	≤1	>4	>8	>8	4
121	≤8	>8	>16	>16	>2	≤1	>4	>8	>8	≤1
122	≤8	>8	16	>16	>2	≤1	>4	>8	>8	2
123	≤8	>8	>16	>16	>2	≤1	>4	>8	>8	2
124	≤8	>8	>16	>16	>2	≤1	>4	>8	>8	≤1

125	≤8	>8	>16	>16	>2	≤1	>4	>8	>8	≤1
126	≤8	>8	>16	>16	>2	≤1	>4	>8	>8	≤1
127	≤8	≤2	>16	>16	>2	≤1	>4	2	8	>4
128	≤8	>8	>16	>16	>2	≤1	>4	>8	>8	2
129	≤8	>8	>16	>16	>2	≤1	>4	≤1	2	4
130	≤8	>8	>16	>16	>2	≤1	>4	2	8	2
131	16	>8	>16	>16	>2	≤1	>4	8	>8	4
132	32	>8	≤1	>16	>2	≤1	>4	>8	>8	4
133	≤8	>8	>16	>16	>2	≤1	>4	>8	>8	2
134	≤8	>8	>16	8	>2	≤1	>4	8	>8	2
135	16	>8	16	>16	>2	≤1	>4	>8	>8	4
136	≤8	>8	>16	>16	>2	≤1	>4	8	4	≤1
137	≤8	>8	16	>16	≤0.5	≤1	>4	4	8	≤1

SI 1 (continued)

SAMPLES	AMI	GEN	CFP	CFO	CIP	COL	ETP	IMI	MER	TGC
138	≤8	>8	>16	>16	>2	≤1	>4	8	2	≤1
139	32	>8	>16	>16	>2	≤1	>4	>8	>8	4
140	≤8	>8	>16	>16	≤0.5	≤1	>4	>8	8	≤1
141	≤8	>8	>16	>16	1	≤1	>4	>8	>8	4
142	≤8	>8	≤1	>16	≤0.5	≤1	>4	>8	>8	≤1
143	≤8	≤2	8	>16	≤0.5	≤1	>4	2	2	≤1
144	≤8	>8	>16	>16	>2	≤1	>4	>8	8	≤1
145	16	>8	≤1	>16	>2	≤1	>4	>8	>8	4
146	32	>8	≤1	>16	>2	≤1	>4	>8	>8	4
147	32	>8	≤1	>16	>2	≤1	>4	>8	>8	4
148	32	>8	>16	>16	>2	≤1	>4	>8	>8	4
149	32	>8	>16	>16	>2	≤1	>4	>8	>8	4
150	32	>8	≤1	>16	>2	≤1	>4	>8	>8	4
151	≤8	>8	≤1	>16	>2	≤1	>4	4	4	>4

152	≤8	>8	>16	>16	>2	≤1	>4	>8	>8	2
153	<8	>8	≤1	>16	>2	≤1	>4	4	>8	2
154	≤8	>8	>16	>16	>2	≤1	>4	>8	8	≤1
155	≤8	>8	>16	>16	>2	≤1	>4	>8	>8	≤1
156	≤8	>8	>16	>16	≤0.5	≤1	>4	8	4	≤1
157	≤8	>8	>16	>16	>2	≤1	>4	8	4	≤1

MIC: Minimum inhibitory concentration; **AMI:** amikacin; **GEN:** gentamicin; **CFP:** cefepime; **CFO:** cefoxitin; **CIP:** ciprofloxacin; **COL:** colistin; **ETP:** ertapenem; **IMI:** imipenem; **MER:** meropenem; **TGC:** tigecycline.
